# Odevixibat as a possible rescue therapy in a pediatric patient with vanishing bile ducts syndrome associated with ethosuximide-induced DILI-DRESS

**DOI:** 10.1093/gastro/goaf014

**Published:** 2025-02-06

**Authors:** Silvio Veraldi, Luca Della Volpe, Valerio Cecinati, Paola Francalanci, Giuseppe Maggiore, Andrea Pietrobattista

**Affiliations:** Division of Metabolic Diseases and Hepatology, Bambino Gesù Children’s Hospital, IRCCS, Rome, Italy; Division of Metabolic Diseases and Hepatology, Bambino Gesù Children’s Hospital, IRCCS, Rome, Italy; Complex Structure of Pediatrics and Pediatric Oncohematology “Nadia Toffa,” Central Hospital Santissima Annunziata, Taranto, Italy; Pathology Unit, Bambino Gesù Children’s Hospital, IRCCS, Rome, Italy; Division of Metabolic Diseases and Hepatology, Bambino Gesù Children’s Hospital, IRCCS, Rome, Italy; Division of Metabolic Diseases and Hepatology, Bambino Gesù Children’s Hospital, IRCCS, Rome, Italy

## Introduction

Idiosyncratic drug-induced liver injury (DILI), particularly with anti-epileptics, occasionally may occur in the setting of severe cutaneous adverse reactions with eosinophilia and systemic symptoms (DRESS), often with a cholestatic pattern of injury, including rarely vanishing bile ducts syndrome (VBDS) [1]. DILI-DRESS can progress from mild damage to fatal cases, with an estimated mortality that ranges from 1.7% to 8% [2]. When VBDS is present, the prognosis is even worse, with unfavorable outcome (liver transplant or death) estimated in 35%–45% of cases [3, 4]. Ileal bile acid transport inhibitors (IBATi) are a new class of drugs that were recently approved for cholestatic pruritus in Alagille syndrome and progressive familial intrahepatic cholestasis, with the aim to reduce the pool of circulating bile acids (BAs) by blocking their uptake from the intestinal lumen [4]. Recently, individual case reports have shown that IBATi use may also be considered for non-conventional indications [5–7].

Herein, we report a case of a 13-year-old boy who was diagnosed with VBDS in the setting of DILI-DRESS, according to the proposed criteria, and unresponsive to all conventional treatments but was successfully treated with odevixibat.

## Case report

**Figure 1.**
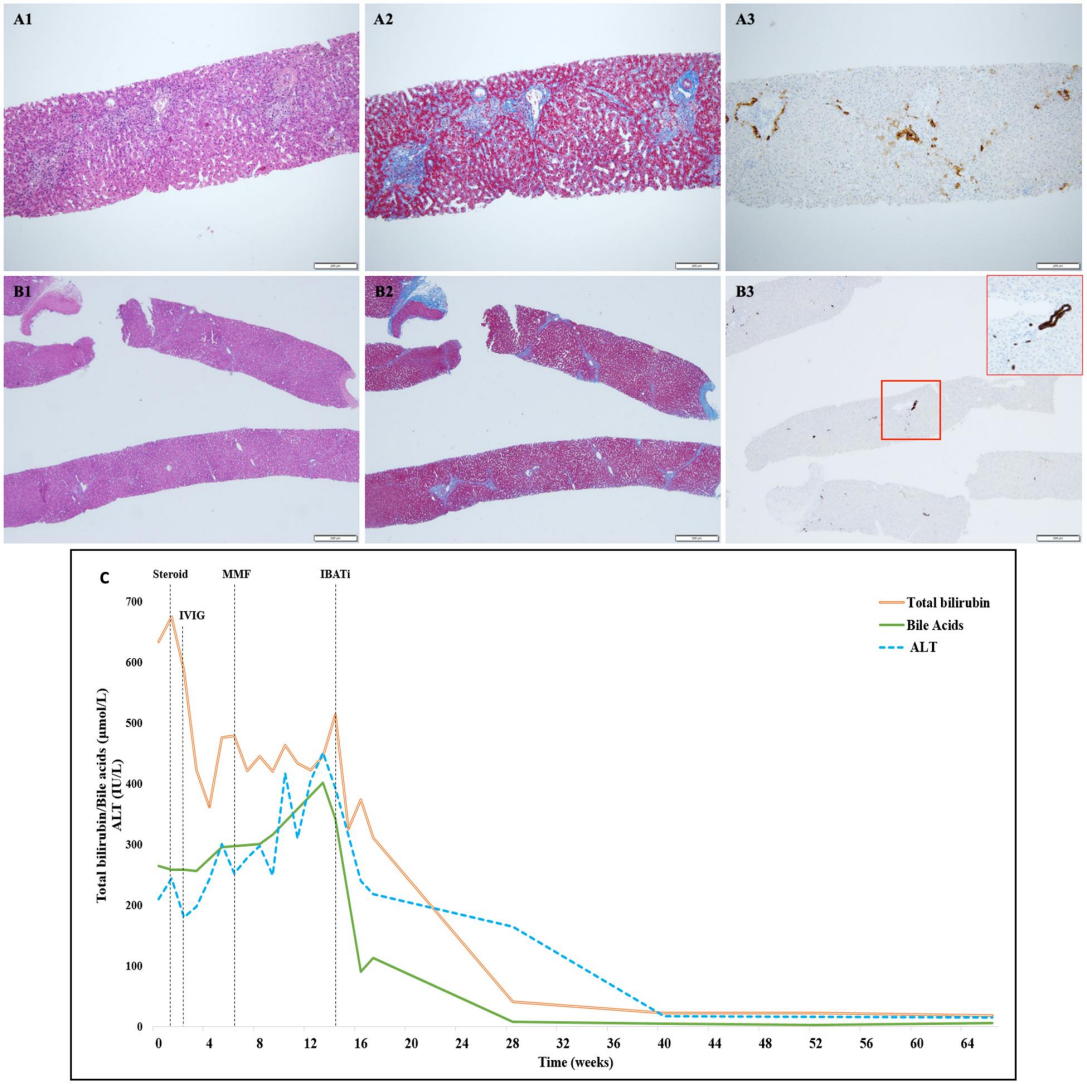
Histopathological assessments (A1–A3) before and (B1–B3) after treatment with odevixibat and a course of laboratory parameters over time (C). (A1–A2) Architecture is preserved. Portal tracts show fibrous expansion and irregular profiles, hematoxylin and eosin, Masson's trichrome, 10×; (A3) immunohistochemistry with anti-CK7 shows loss of the interlobular bile duct with periportal ductal metaplasia, CK7, 10×. (B1–B2) Architecture is preserved with portal tracts preserved in size and morphology, hematoxylin and eosin, Masson's trichrome, 4×; (B3) the interlobular bile duct is evident in all portal tracts, CK7, 4× and 20×. IBATi = ileal bile acid transporter inhibitors, IVIG = intravenous immunoglobulin, MMF = mycophenolate mofetil.

The patient was under neurologic follow-up for myoclonic–atonic epilepsy (OMIM: #616421)—a neurodevelopmental disorder with seizures. Because of poor seizures control, ethosuximide was added to the previous anticonvulsant treatment (clobazam and valproate). However, 2 weeks later, the patient developed fever, jaundice, and a maculo-papular rash, and therefore was referred to a local hospital. Laboratory tests showed eosinophilia (eosinophils: 1.940/mm^3^) and cholestatic hepatitis with preserved liver synthetic function. Ultrasound and CT scan revealed mild hepatosplenomegaly with no signs suggestive of anomalies of the biliary tree. Previous personal and familial medical history was negative for liver diseases. Ursodeoxycholic acid and empiric antibiotic treatment were started while ethosuximide was promptly discontinued. Due to the worsening of the cholestasis (total bilirubin: 630 μM/L, direct bilirubin 476 μM/L, BAs: 264 μM/L), the patient was referred to our Pediatric Hepatology Center. At the admission, an extensive diagnostic workup for acute and chronic cause of liver disease was carried out, resulting as negative (e.g. autoimmunity, ceruloplasmin, alpha-1-antitrypsin, 24-h urinary copper, full microbiological screening, metabolic investigations). Considering the recent introduction of ethosuximide, the 2016 updated RUCAM score was calculated as being 5, in keeping with a possible DILI [8]. The patient underwent a magnetic resonance cholangiopancreatography, excluding signs of extrahepatic cholestasis. Acoustic radiation force impulse (ARFI) imaging revealed a raised hepatic stiffness of 11 kPa, which increased to 14 kPa after 4 weeks. Clinically, cholestatic itch was present so rifampicin was initiated but proved to be ineffective. A clinical exome was then performed through next-generation sequencing, focusing on all genes that are primarily or potentially involved in liver diseases, but no pathogenic variants were identified, even at the state of haploinsufficiency. The patient underwent a liver biopsy with histology showing signs suggestive of VBDS (Figure 1A1–3). The patient was treated with steroids (methylprednisolone, 60 mg/day) and a single immunoglobulin infusion (800 mg/kg), with only partial and temporary response. In the following weeks, a novel increase in bilirubin was observed and a second immunosuppressant was added (mycophenolate mofetil). Moreover, malnutrition became evident (BMI 13.3 kg/m^2^, z-score −3.61) requiring artificial nutritional support via a nasogastric tube. Fourteen weeks after the onset of liver involvement, a second histological evaluation was performed that confirmed VBDS with further progression of fibrosis. Because of evidence of treatment failure, the steroid and mycophenolate were gradually reduced until suspension. At that time, liver tests showed total bilirubin and direct bilirubin of 470 and 360 μM/L, with BAs of 342 μM/L, alanine aminotransferase (ALT), aspartate aminotransferase, and gamma-glutamyl transferase were 293, 312, and 1,061 IU/L, respectively. Regular ultrasound monitoring revealed a micro- and macro-nodular aspect of the right lobe of the liver. Considering the scenario of severe and progressive intrahepatic cholestasis associated with significant itch in a patient with a complex neurological disorder, we opted for a rescue therapy with odevixibat (40 mcg/kg/day). Following treatment initiation, we experienced a gradual but steady improvement in cholestatic parameters and clinical symptoms, noteworthy pruritus, and nutritional status (BMI 15.2 kg/m^2^, z-score −1.85). Complete liver test normalization was achieved in 4 months (Figure 1C) and was maintained thereafter while no drug-related adverse reactions were ever recorded. At 12 months after starting odevixibat, a repeated liver biopsy showed restoration of near-normal tissue architecture and complete VBDS resolution (Figure 1B1–3). Liver ultrasound confirmed the reversion of the nodular aspects of the right lobe while the ARFI decreased to 3.9 kPa. Considering all these data, we decided to discontinue odevixibat. To date, 6 months after suspension, the patient is in good clinical condition with normal liver function tests and no seizures were observed after switching ethosuximide for levetiracetam.

## Discussion

Odevixibat was revealed to be an effective strategy in progressive cholestatic liver injury in the setting of refractory DILI-DRESS. The concurrence in time of treatment initiation and prompt improvement in the patient’s biochemical, radiological, and clinical features might indicate a major role for odevixibat in VBDS resolution. Decades of research have provided insights into the role of BA in cholestatic liver injury and abnormal BA homeostasis is now known to be involved in the complex pathophysiology of DILI [9, 10]. Even if BAs do not represent the primary cause of cholestatic injury, their accumulation affects each cellular component of the liver: hepatocytes, cholangiocytes, stellate cells, and immune cells. Pruritus is known to be associated with chronic or acute cholestatic liver disease, such as DILI. The mechanism of pruritus in cholestasis is not completely understood; however, it is believed to be largely secondary to increased circulating levels of pruritogens, such as BAs. It can significantly affect quality of life, particularly when refractory to standard treatments and, as reported, also in some cases of DILI that require more invasive treatment, such as plasmapheresis.

We might speculate that the rapid pharmacological reduction in the intrahepatic BA load may have interrupted the inflammatory, cytotoxic, and fibrotic damage, thus allowing the resolution of the liver injury process and, consequently, itching. Further studies are advocated to assess the effectiveness of IBATi and patient eligibility in expanded indications other than genetic cholestatic diseases.

## Authors’ Contributions

Conceptualization and writing original draft paper: A.P., L.D.V., S.V., P.F. and V.C. Conceptualization, review and editing of the paper: A.P., G.M. Final approval of the version: all the authors.
